# Death by transposition – the enemy within?

**DOI:** 10.1002/bies.201300097

**Published:** 2013-10-15

**Authors:** John M. Sedivy, Jill A. Kreiling, Nicola Neretti, Marco De Cecco, Steven W. Criscione, Jeffrey W. Hofmann, Xiaoai Zhao, Takahiro Ito, Abigail L. Peterson

**Affiliations:** Department of Molecular Biology, Cell Biology and Biochemistry, Brown University, Providence, Rhode Island, USA

**Keywords:** aging, anti-retroviral therapy, cellular senescence, retrotransposition

## Abstract

Here we present and develop the hypothesis that the derepression of endogenous retrotransposable elements (RTEs) – genomic parasites – is an important and hitherto under-unexplored molecular aging process that can potentially occur in most tissues. We further envision that the activation and continued presence of retrotransposition contribute to age-associated tissue degeneration and pathology. Chromatin is a complex and dynamic structure that needs to be maintained in a functional state throughout our lifetime. Studies of diverse species have revealed that chromatin undergoes extensive rearrangements during aging. Cellular senescence, an important component of mammalian aging, has recently been associated with decreased heterochromatinization of normally silenced regions of the genome. These changes lead to the expression of RTEs, culminating in their transposition. RTEs are common in all kingdoms of life, and comprise close to 50% of mammalian genomes. They are tightly controlled, as their activity is highly destabilizing and mutagenic to their resident genomes.

## Introduction

The steep increase in life expectancy in the 20th century, enabled by modern clinical and public health interventions, is a remarkable phenomenon in human history. Aging is a fundamental biological process with a profound impact on medicine and society. Age-related disorders, such as cardiovascular diseases, stroke, diabetes, and cancer have become the leading causes of death in developed countries. The incidence of all these, and many other debilitating diseases such as dementia, shows a near exponential increase in the final third of human life span [1].

Our understanding of the biology of aging has also made an important shift, as conserved mechanisms have been identified that modulate aging in diverse species. The notion that age-related illnesses are solely influenced by a multitude of independent genes and/or behavioral risk factors has been dispelled by research showing that simple genetic and dietary interventions can retard many late-life diseases in parallel [2–4]. In other words, in addition to disease-specific processes, basic and universal mechanisms have been identified that influence organismal aging and impact virtually all organ systems. The therapeutic potentials are immense – and pharmacological interventions have already achieved impressive life span extensions in model organisms, including the mouse [5].

The compression of morbidity is of particular interest, since an increasing number of reports document amelioration of a variety of aging phenotypes without significant life span extension [6]. This has focused attention on health span – the fraction of our lives free of frailty and chronic disorders – as a realistic and desirable immediate goal. For example, resveratrol treatment of mice and calorie restriction of rhesus monkeys have already shown a range of physiological benefits, without achieving, as yet, clear longevity effects [7–9]. It is very likely that further research will yield clues for additional targets of pharmacological intervention.

Many hypotheses have been posited to explain the molecular aspects of aging. Most models have one common theme: the accumulation of molecular damage that results in a subsequent deterioration of cellular and tissue structure and function. These mechanisms are not mutually exclusive, and important points of crosstalk have already been found. In this paper we develop the hypothesis that the activation of transposable elements (TEs), which inhabit the genomes of all life forms, is an important and hitherto poorly explored molecular aging process. Transposition is known to be highly mutagenic, and we propose that the ensuing genomic destabilization may be an important driver of cellular and tissue dysfunction.

## Cells can age chronologically as well as replicatively

Aging occurs at virtually every level of complexity – from molecules to tissues to the whole organism. Two fundamental cellular aging processes have been described. One is chronological aging, and is the consequence of time-dependent changes that occur independently of cell division. It is thus of paramount importance in terminally differentiated cells. An important component of chronological aging is a breakdown of the balance between biosynthesis, repair and turnover, leading to the accumulation of damaged or otherwise dysfunctional macromolecules [10, 11]. Replicative aging, on the other hand, reflects the ability of a cell (and its lineage) to support ongoing rounds of cell division, and is of major importance in organisms whose adult bodies depend on extensive tissue turnover. Adult stem cells that support the regeneration of most of our tissues and organs are believed to be at significant risk of both chronological and replicative aging [12].

## Cellular senescence contributes to aging

Replicative cellular senescence was first described as an irreversible arrest of proliferation triggered by the accumulation of cell divisions. The underlying cause of senescence due to replicative exhaustion is telomere shortening. In the last decade it became evident that what was classically described as replicative senescence is in fact a collection of interrelated states that can be triggered by distinct intrinsic and extrinsic stimuli [13]. Many types of stress, including reactive oxygen species (ROS) and activation of oncogenes, can trigger a senescence response [14].

While numerous studies have implicated cellular senescence as an important in vivo tumor suppression mechanism [15], convincing evidence for a role in organismal aging has been slow to emerge [16]. One impediment has been the lack of reliable assays to distinguish senescent cells from the majority of healthy but quiescent cells in normal tissues. The first useful biomarker was the senescence-associated β-galactosidase [17], but the lability of its activity can result in high false-negative rates. A fluorescence microscopy method to visualize telomeric double-strand breaks (DSB) in intact nuclei [18], often referred to as the telomere dysfunction-induced focus (TIF) assay, has proven to be a reasonably robust biomarker. Examination of dermal biopsies collected from baboons of various ages showed an exponential rise of telomere dysfunction with age, affecting 15–20% of all cells in old animals [19]. Using a different marker of senescence, the cyclin-dependent kinase (CDK) inhibitor p16^Ink4a^, it was found that humans genetically predisposed to extreme longevity display reduced frequencies of senescent cells [20]. In a landmark paper, the van Deursen group showed that in vivo pharmacological ablation of tissue-resident senescent cells in a progeroid mouse model provided clear health span benefits [21]. Hence, there is much current interest in the targeting of senescent cells as a possible therapy for age-associated diseases [22].

## Chromatin is regulated epigenetically and changes in senescence and aging

It has long been appreciated that an important aspect of aging may be biological structures that are inherently difficult to maintain [23, 24]. Chromatin is one such structure, and it is rapidly becoming clear that it is subject to extensive age-associated remodeling [10]. Changes in the organization of the genome into euchromatin and heterochromatin, as well as in DNA methylation patterns during aging and cellular senescence have been documented in several species [25, 26]. For example, a decrease in histone expression has been noted in senescent cells [27, 28], and histone overexpression can extend the lifespan of yeast [29]. In the nematode, preventing excess accumulation of histone activating marks (H3K4Me3) extended lifespan [30]. Mammalian cells undergoing senescence reorganize their genomes into prominent senescence-associated heterochromatin foci (SAHF) [31]. Historically, changes in the methylation of CpG residues in DNA were the first noted effects of aging on chromatin [32, 33], and have been widely studied [34, 35]. While overall levels of DNA methylation decrease, methylation of CpG islands associated with promoters (and hence transcriptional regulation of genes) changes in complex and tissue-specific patterns, showing an overall increase in many cases.

Using high throughput sequencing approaches, we recently examined genome-wide changes in chromatin accessibility that occur during replicative cellular senescence [36]. We found that the fundamental architecture of the genome undergoes profound alterations: an overall closing of chromatin in euchromatic gene-rich regions, which is opposed by a somewhat paradoxical opening of heterochromatic gene-poor regions. The former was associated with a dampening of gene expression, and the latter with increased transcription of retrotransposable elements (RTEs, below), which are normally heavily heterochromatinized. Quite remarkably, this culminated in active transposition, as evidenced by increases in copy number. Heterochromatin in centromeric and pericentromeric regions also became more open, and transcription of satellite sequences increased.

## Transposable elements are genomic parasites

Mobile genetic elements, also known as TEs, were discovered by Barbara McClintock in maize, and have since been found to be ubiquitous in all domains of life [37]. They are commonly viewed as molecular parasites that inhabit and at times move in the genomes of their hosts, with mostly deleterious consequences. One group of TEs, referred to as DNA transposons, move from one genomic location to another by a “cut-and-paste” mechanism. Another group, known as RTEs, use a “copy-and-paste” mechanism: an RNA copy of the element is first made by a cellular RNA polymerase, then converted back into DNA by a reverse transcriptase enzyme encoded by the element, and finally inserted into a new genomic location. RTEs are a large and diverse group, and include retroviruses [e.g. human immunodeficiency virus (HIV)], long terminal repeat (LTR) RTEs (e.g. yeast Ty1, or *Drosophila* Copia), long interspersed nuclear elements (LINEs, e.g. mammalian L1), and short interspersed nuclear elements (SINEs, e.g. human Alu). Some retroviruses have extracellular phases in their life cycles, but other elements are obligatorily intracellular. About half of mammalian genomes (by some recent estimates, maybe as much as two-thirds [38]) consist of a variety of TEs, the great majority being RTEs.

Organisms counteract the transposition of RTEs by repressing their activity at several levels, the primary line of defense being heterochromatinization of the genomic regions where they are inserted [39]. This prevents their transcription, and hence expression of the reverse transcriptase and other activities needed for transposition. Being mostly passive passengers in the genomes of their hosts, RTEs are subject to genetic drift, and the great majority of RTEs gradually accumulate mutations or rearrangements that render them incapable of transposition. The only RTE believed to be currently autonomously active in humans is the LINE L1 [40]. It comprises ~17% of our genome (~500,000 copies), but only members of the evolutionarily recent and human-specific subfamily, L1Hs (and to a lesser extent, members of the somewhat older primate L1PA subfamilies) are believed to be active [41, 42]. There are ~1,600 known members of the L1Hs subfamily, of which some 200–300 appear to be intact and hence potentially active. These values are likely to be revised upward, as the L1Hs subfamily remains incompletely annotated in the hg19 build of the genome. All SINEs are non-autonomous as they do not encode any proteins, but their movement can be catalyzed in *trans* by the enzymes encoded by LINEs [43]. Both L1s and their SINE “parasites” show considerable activity in human germline genomes, estimated at one new L1 insertion in 95–270 births and one new Alu insertion in ~20 births [44]. A large fraction of human polymorphisms are due to RTEs, and many disease-causing insertions have been identified [40, 45]. In mouse, over 15% of spontaneous mutations in laboratory strains have been estimated to be caused by RTEs [37, 46].

Much of our accumulated knowledge of RTE biology reflects their activity in the germline, since transposition events in somatic tissues would not be inherited from generation to generation, and hence would not be captured in reference genome sequences. Recent evidence indicates that retrotransposition in the soma may be much more active than previously believed, especially during early embryogenesis, and also appears to occur frequently during cancer development [42, 44, 47, 48]. Disruption of tumor suppressor genes (by insertional mutagenesis) and inappropriate activation of positive effectors (by epigenetic dysregulation) are obvious mechanisms that could promote tumorigenesis, however, the extent to which activation of retrotransposition contributes to the total human cancer burden is currently actively debated [49]. One tissue where retrotransposition appears to be especially active is the nervous system: L1 mobilization was observed in neural progenitor cells during development as well as adult neurogenesis, and frequent new L1 insertions were found in adult brain tissue relative to other tissues [50, 51]. Hence, L1 transposition may be “allowed” in a specific window during neurogenesis as a mechanism of somatic diversification that contributes to neuronal plasticity [52], along the lines that somatic hypermutation and rearrangement contribute to immune diversity. These ideas however remain controversial, as a recent study did not find evidence for frequent L1 insertions in the brain [53].

## Is retrotransposition activated during aging?

L1 transposition in nondividing cells has been reported to be either low or absent, but was clearly detectable in fully differentiated postmitotic cells, such as hepatocytes, in cell culture [54, 55]. Our recent data demonstrate that L1 copy number increases in senescent fibroblasts [36]. While we cannot rule out that mobilization was initiated while some cells were dividing, copy number continued to increase at later times after all replication ceased. In recent unpublished studies we noted further increases (up to 40%) in the copy number of L1Hs and some L1PA subfamilies in fibroblasts that were senescent for extended periods of time (>6 months). In other examples, the *S. cerevisiae* LTR RTE Ty1 is mobilized during chronological aging of this species [56], several *D. melanogaster* LINE and LTR RTEs become active in aging neurons [57], and a single novel IAP insertion in the mouse was noted to become more active with age [58]. Hence, the age-associated “loosening” of RTEs appears to be a remarkably conserved phenomenon.

Senescent cells differ from normal postmitotic cells in that they are in a state of chronic DNA damage [59]. It is well documented that transcription of RTEs and other repetitive elements (such as satellites) is activated by DNA damage, as well as a variety of other stresses including heat shock and ROS [60–63]. L1 copy number increases were found to be augmented in brain tissue of ataxia telangiectasia patients, a syndrome associated with increased and persistent DNA damage [64]. It is hence quite reasonable that the transcription of RTEs becomes activated in senescent cells, and our data show there are no significant blocks to transposition in the senescent state, especially over long periods of time.

Conventional wisdom has been that only replicatively competent cells can enter senescence, but this view probably needs adjustment. As the many phenotypes of senescent cells have come into sharper focus, it is now evident that even postmitotic cells such as neurons or skeletal muscle cells can acquire some of these features [65, 66]. Postmitotic cells could enter a senescent state by acquiring irreparable telomeric damage, which can occur even without critical telomere erosion (and may be caused by a variety of stresses, including ROS) [67, 68]. Irreparable non-telomeric DNA damage has also been noted [69]. These studies have important implications, as they suggest that cellular senescence could afflict not only relatively few cells that have very short telomeres or activated oncogenes, but in principle any cell in the body (given appropriate and sufficient stresses as trigger).

The activation of RTEs in postmitotic cells could also proceed through mechanisms that do not involve DNA damage or genotoxic stress. For example, it has been known for some time that overall genomic DNA methylation decreases during aging, and that this occurs largely in repetitive DNA sequences [26]. Methylcytosine residues are bound by methyl-CpG-binding protein 2 (MeCP2), which then recruits histone deacetylases and other chromatin regulators to promote heterochromatinization and inhibit transcription. Neuronal L1 retrotransposition is increased in MeCP2 knockout mice as well as in Rett syndrome patients that carry MeCP2 mutations [70]. The expression of the DNA (cytosine-5)-methyltransferase 1 (DNMT1) is also known to decrease in cells approaching senescence in culture [71], but why this may happen, and what other factors may contribute to the decrease of DNA methylation in vivo is not well understood.

Expression of RTEs is also opposed by several small noncoding RNA mechanisms, including miRNA, siRNA, and piRNA pathways that act to silence transcription, promote mRNA destruction, or inhibit translation [72, 73]. For example, the piRNA pathway is critical for RTE silencing and genome maintenance in the *Drosophila* and mouse germline [74–76], and recent evidence suggests that changes in its activity may allow transposition in adult somatic tissue [77]. siRNAs have been implicated both in *Drosophila* and mouse [78, 79], and L1-specific small RNAs were noted in human somatic cells [80]. Defects in the miRNA pathway have been noted during aging in some mouse tissues [81], and implicated in allowing RTE activity during cancer development [82].

The biology of TEs and RTEs has been intensely studied in invertebrate model systems, including plants, fungi, and bacteria, but much remains to be learned about their behavior in mammals. It is quite evident, however, that their activity is opposed at multiple levels, that these mechanisms have potentially numerous vulnerabilities, and that the consequences of allowing an RTE onslaught are likely to be profoundly deleterious. For example, mutations in the DNA methylase Dnmt3L in mice, or components of the piRNA pathway in *Drosophila* or mice, allow the expression and mobilization of RTEs in the germline of sufficient magnitude to cause meiotic failure and sterility [40, 74, 83, 84].

Transposition of both DNA transposons and retrotransposons can also have beneficial effects: e.g. by promoting new patterns of gene expression [85], and even contributing to evolutionary bursts and speciation [86]. This has engendered a long-standing debate on whether the activity of TEs and RTEs is intrinsically “good” or “bad”, and the possible relationships and trade-offs between these concepts [87, 88]. Others have argued that at the level of a single individual, these processes, and especially those occurring in somatic tissues, are likely to be overwhelmingly deleterious [40, 89].

Somatic retrotransposition in mammals, in particular, remains a little explored area, simply because until recently we lacked the tools to adequately document the activity of these endogenous elements. High throughput sequencing is changing all this, but because of the highly repetitive nature of the datasets, comprehensive annotation remains a very challenging task. There is widespread anticipation however that what has come to light so far is just the tip of the iceberg.

## Formulation of the “aging by transposition” hypothesis

To allow a critical evaluation of the rationale and potential impact of our proposal, we first summarize the key points of our current knowledge: (i) global regulatory networks have been found that affect the rate of organismal aging; (ii) these networks can be manipulated genetically and pharmacologically to positively impact health span; (iii) replicative cellular senescence has emerged as an important factor in age-associated pathologies; (iv) we have discovered that cellular senescence leads to a dramatic activation and mobilization of RTEs, which is a new and hitherto unknown molecular phenotype of cellular senescence; (v) recent studies have uncovered unifying connections between chronological and replicative cellular aging, indicating that virtually all cells in the body may have the capacity (if appropriately triggered) to enter into a dysfunctional state resembling in some aspects cellular senescence.

**Figure 1:**
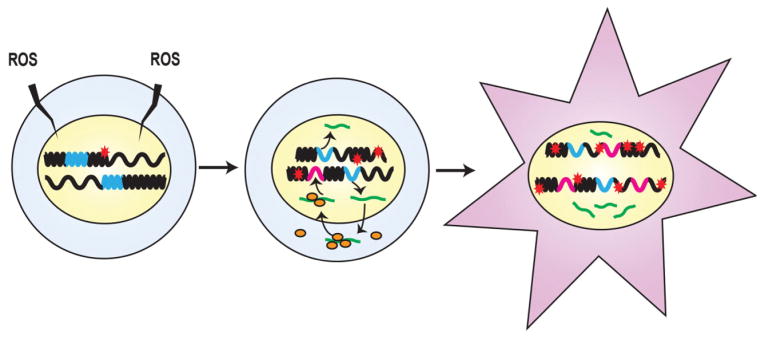
Illustration of the “aging by transposition” hypothesis. A *normal cell* is shown on the left. Heterochromatic (densely wound) and euchromatic (loosely wound) regions of chromosomes (black) are shown. RTEs (blue) are located mostly in heterochromatin and are not expressed. ROS (and other stresses) cause sporadic DNA damage (red star), which is mostly repaired. A *compromised cell* is shown in the middle. Increasing stress (not necessarily limited to DNA damage) is starting to promote a redistribution of chromatin. Some heterochromatic regions are becoming more open, and this leads to increasing expression of RNA from the resident RTEs (shown as green lines). The RTE mRNAs (green) migrate to the cytoplasm, where some are translated into encoded ORF1 and ORF2 proteins (orange ovals), which subsequently assemble with the mRNAs into RTE nucleoprotein particles. Some nucleoprotein particles migrate back to the nucleus, where they are reverse transcribed and finally insert into new genomic locations (shown as magenta). Abortive retrotransposition events promote new DNA damage (more red stars). A *dysfunctional* cell is shown on the right. Continuing expression of RTEs, ongoing transposition, and accumulating DNA damage drive widespread chromatin rearrangements, genomic instability, changes in gene expression, and mutagenesis.

On the basis of this analysis we propose the following hypotheses: (i) activation of RTEs is a common feature of cellular senescence; (ii) the mobilization of RTEs is an important contributor to the deleterious phenotypes of senescent cells; (iii) somatic retrotransposition is a widespread age-associated process that can affect many tissues and could afflict both replicative and postmitotic cells. Possible connections between RTE activity and aging have also been noted, on a theoretical basis, by others [88, 89]. A simplified scheme for what we envision as a generalized and reasonable sequence of these events is depicted in Fig. 1.

**Figure 2:**
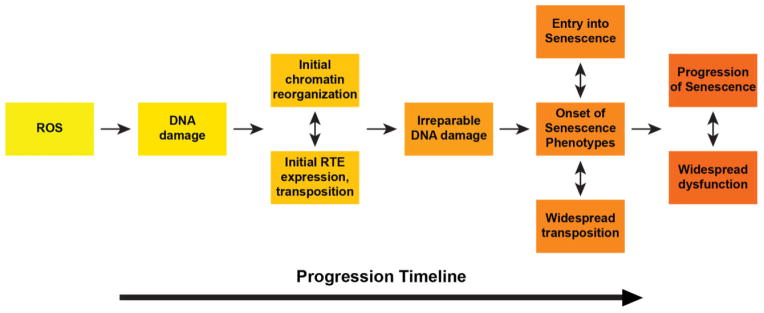
A schematic showing in more detail one possible sequence of events from an initiating stress to the activation of retrotransposition, cellular senescence, and ultimately tissue pathology. In this example ROS is again used as the initiating stress and the activation of RTEs is envisioned to occur relatively early. By promoting widespread DSBs, the expression of L1 proteins accelerates the generation of irreparable DNA damage. This acts as a trigger for cellular senescence, which in turn promotes tissue dysfunction. However, the events depicted here could be arranged in different combinations, and we know of no mechanistic considerations that would a priori eliminate many of these alternatives. For example, retrotransposition could also be initiated at later times, such as during the establishment or even progression of senescence. Furthermore, initiating events other than ROS and DNA damage could lead to the activation of RTE expression.

However, numerous alternative scenarios for this RTE “jail break” can be envisioned (see Fig. 2 for a more detailed schematic representation). For example, RTE mobilization could be a relatively late event, or occur too rarely to make a significant contribution to the tissue damage elicited by senescent cells. Conversely, it could play an important role in the initiation, progression, or overall negative effects of cellular senescence. Intermediate situations are also plausible, namely, that the frequency, time line, and downstream consequences of transposition may vary with the triggering event, cell type, tissue, or some other parameter. In considering the possible time lines (Fig. 2), it is important to note that the mutagenic and destabilizing effects of retrotransposition are not solely due to insertions into new genomic locations. It has been shown that transposition is accompanied by a significant number of incomplete or abortive events that result in the creation of widespread DSB throughout the genome [90, 91]. In cell culture experiments it is these DSB, and not bona fide transposition, that have been found to trigger premature senescence [90]. We can thus envision the potential for considerable positive feedback loops, given that, as noted before, transcription of RTEs and other repetitive elements is activated by DNA damage [60–63].

Is the activation of retrotransposition then a cause or effect of cellular senescence, and what are the connections with aging? The interpretation most consistent with available data is that retrotransposition is an effect of cellular senescence (but this may of course change as we learn more). We know now that cellular senescence involves the derepression of RTEs [36], which we suggest then contribute to the aging decline. The latter could involve a number of mechanisms. The most obvious, for which there is considerable experimental evidence (as discussed above), is the promotion of DNA damage and genome instability. Whether the senescence-associated secretory phenotype (SASP [92, 93]) is impacted in any way by RTE mobilization has not been examined. It has been suggested that the production of RTE mRNA and cytoplasmic ribonucleoprotein particles could trigger an antiviral response or other aspects of innate immunity [94]. The activation of resident intergenic RTEs could have impacts on the normal regulation of gene expression [85], as of course could new insertions throughout the genome. Finally, these events could lead to the redistribution of chromatin regulators and consequent large-scale changes in chromatin organization, already hinted at by the loss of constitutive heterochromatin at centromeres [36]. Another important precedent for age-associated changes in chromatin organization is the redistribution of SIRT1 protein to sites of DNA damage [95].

We also note further extensions of the most parsimonious interpretation (above) of RTE mobilization as an effect of cellular senescence. First, RTEs could be derepressed independently of replicative senescence, in chronologically aging cells. This is raised by recent data, noted above, showing that chronologically aging postmitotic cells can acquire some phenotypes of senescent cells, and that the chromatin of chronologically aging cells can undergo changes that could affect RTEs. Second, if RTEs are derepressed by mechanisms independent of senescence, their mobilization (by promoting DNA damage) could in turn induce senescence.

In aggregate, these considerations suggest that inhibiting RTE mobilization may have therapeutic benefits. Given the uncertainties discussed above, such interventions may affect only a subset of age-associated pathologies, or only in some tissues. Such an outcome, however, could still be very positive in managing certain aspects of health span in the clinic. While Alu and SVA are the most active elements in the human genome, L1 is the real culprit, since it encodes the enzymatic machinery that the SINEs exploit. Management of L1 LINEs would thus be a good starting point for manipulation, but other elements and possible mechanisms of pathology should also be noted [94].

## Testing the hypothesis: Retrotransposition can be inhibited pharmaceutically

The next obvious question that comes to mind after considering this scenario is: Can retrotransposition be inhibited or controlled? Viruses that incorporate reverse transcription as part of their life cycles, such HIV or hepatitis B virus (HBV), are a major healthcare problem, and enormous efforts have been focused on developing drugs to treat these infections. The first successful drugs, still widely used today, were nucleoside and nucleotide analog reverse transcriptase inhibitors (nRTIs, ntRTIs). These drugs bind to the catalytic site of reverse transcriptase and competitively inhibit its polymerase activity. Development of these drugs has gone through several generations of improvements and extensive safety and efficacy testing. The major off target effects are due to interactions with other cellular polymerases, in particular mitochondrial DNA polymerase *γ* (encoded by the nuclear *POLG* gene) [96].

Early efforts to control HIV with first generation nRTIs and ntRTIs resulted in rapid emergence of resistance and recurrence of disease. This was due to the high mutation rate of HIV and the subsequent multiplication and spread of the drug-resistant viruses. Current strategies, which have been very successful in controlling HIV, employ combinatorial therapy based on multiple drugs that inhibit a number of viral activities, including viral protease inhibitors, integrase inhibitors, and non-nucleoside inhibitors of reverse transcriptase. nRTIs and ntRTIs remain in wide use as important components of these combinatorial therapies.

Given these precedents, it is straightforward to envision the development of nRTI and ntRTI inhibitors specific for the human LINE L1 reverse transcriptase, the only endogenous RTE that is believed to be currently active in the human genome. Such drugs would have to be screened, as has been done in the past, for off target effects on other cellular polymerases. Remarkably, several (but by no means all) existing nRTIs and ntRTIs that are in use to treat viruses such as HIV and HBV also effectively inhibit the reverse transcriptase of a variety of unrelated RTEs, including murine retroviruses, human and mouse L1s, and yeast Ty1 elements [54, 97, 98]. These findings are quite serendipitous, as these drugs were never intended (or screened) for inhibition of L1 reverse transcriptase.

One drug in particular, lamivudine, which is currently widely used for the treatment of HBV and as part of combinatorial therapy for the treatment of HIV-AIDS, is a potent inhibitor of human L1 reverse transcriptase (with a *K*_i_ of 12.9 ± 2.07 nM) [97]. In human cell culture assays, using engineered L1 reporter elements, lamivudine inhibited retrotransposition by 92–97% [97]. Given that lamivudine also inhibits mouse L1 reverse transcriptase, proof-of-principle experiments, using the mouse as the model system, can be started right away to investigate efficacy against a large variety of age-associated pathologies. Prior to its approval for human use, lamivudine was extensively tested in mice (and rats), and is well tolerated at doses much higher than those used for human therapy.

It is also worth noting that the key issue with the emergence of drug-resistant viruses is their rapid spread throughout the infected organism. It can be anticipated that this will be an issue of much smaller proportions with endogenous RTEs, as they are obligatorily intracellular. For example, in the case of cancer development, even if resistant L1 variants arose at rates similar to those documented in HBV and HIV, effective RTI therapy would be expected to reduce the number of cells experiencing genetic instability by a factor of 1,000–10,000-fold.

## Conclusions and prospects

In this essay we have developed the concept that retrotransposition in somatic tissues is a force of genomic structural dysregulation that acts as a driver of aging-related phenotypes. What sets these forces in motion we do not know, but we propose that this deficit in genome management is one of the fundamental causes of aging.

We need to learn much more about the behavior of our endogenous RTE parasites in our somatic tissues before future therapeutic applications can be assessed with any accuracy. We especially need to learn about the forces that shape our genomes with advancing age and allow the RTEs to become active during cellular senescence, or perhaps, chronological aging of some cells. Given the availability of drugs like lamivudine, we can also immediately begin testing the consequences of inhibiting L1 reverse transcriptase, both in cell culture and in the mouse. Much will depend on how the “time line” of L1 activation and its consequences (Figs. 1, 2) emerges from further research. As pointed out above, these effects could vary quite considerably between different tissues and for different pathologies, so they will have to be investigated in some detail. In addition, as already noted, even in the case of relatively “late” events, there may be considerable merit in alleviating some symptoms of associated chronic pathologies.

In trying to envision a “best case” scenario, we should consider the already demonstrated activity of L1 RTEs during cancer development, and in the nervous system. For the former, given the known role of genetic instability in promoting cancer, and the known role of RTEs in promoting genetic instability, the possible association is obvious. For the latter, it is believed that ROS may drive some of the proteotoxicities associated with Alzheimer’s and other dementias, and ROS is known to activate the transcription of some RTEs. Although this association is more tenuous, it is well within reason that activation of retrotransposition and ensuing genomic instability could contribute to age-associated neuronal degeneration. Hence, optimistically, we could be looking at better prevention and management of cancer and dementia, two of the most debilitating age-associated human conditions.

## Abbreviations

CDK: cyclin-dependent kinase
DNMT1: DNA (cytosine-5)-methyltransferase 1
DSB: double-strand breaks
H3K4Me3: histone H3 lysine 4 trimethylated
HBV: hepatitis B virus
HIV: human immunodeficiency virus
LINE: long interspersed nuclear element
LTR: long terminal repeat
MeCP2: methyl-CpG-binding protein 2
nRTI: nucleoside reverse transcriptase inhibitor
ntRTI: nucleotide reverse transcriptase inhibitor
ROS: reactive oxygen species
RTE: retrotransposable element
SAHF: senescence-associated heterochromatin foci
SASP: senescence-associated secretory phenotype
SINE: short interspersed nuclear element
TE: transposable element
TIF: telomere dysfunction-induced focus
